# O.6.2-7 “Virtual Surf Booth”: assessment of a novel tool and data collection process to measure the impact of a 6-week surf programme on wellbeing

**DOI:** 10.1093/eurpub/ckad133.277

**Published:** 2023-09-11

**Authors:** Ariane Gerami, Charlie Foster, Joey Murphy

**Affiliations:** Centre for Exercise, Nutrition and Health Sciences, School for Policy Studies, University of Bristol, UK; ariane.gerami@gmail.com; Centre for Exercise, Nutrition and Health Sciences, School for Policy Studies, University of Bristol, UK; ariane.gerami@gmail.com; Centre for Exercise, Nutrition and Health Sciences, School for Policy Studies, University of Bristol, UK; ariane.gerami@gmail.com

## Abstract

**Purpose:**

Practicing physical activity in blue space (i.e. forms of natural and manmade surface water) is shown to be associated with an improvement in wellbeing. Surfing is a popular blue space activity with surf therapy increasingly used as a health intervention. However, the mechanisms through which surfing leads to an improvement in wellbeing remain unclear. One reason for this is the challenges with collecting data related to acute and chronic wellbeing outcomes. The purpose of this study was to evaluate the usability of an online digital tool to measure wellbeing outcomes, and the usefulness of the data collected.

**Methods:**

Participants of a 6-week surf programme, delivered at The Wave Bristol, were invited to partake. Each participant was asked to complete the online digital tool on weeks 1, 2, 4 and 6. Qualitative (video recording and word association) and quantitative (Short Warwick-Edinburgh Mental Well-Being scale) responses were collected. Usability and acceptability of the tool were evaluated through focus groups with participants and engagement data. Descriptive analysis and paired samples t-tests were used to assess differences in wellbeing during the surf programme. A reflexive thematic analysis was used to assess video and word association responses.

**Results:**

Participants (n = 15, 100% female, age=43.0±5 years) perceived the tool as easy to use due to the short completion time, range of tool functionalities, and useful because it provided time for reflection. Aspects such as increasing privacy when using the tool, accessibility of the tool, and incentivisation with completing the questions were seen as aspects that can increase future engagement. From a research perspective, the data collected allowed for the assessment of acute and chronic wellbeing, and demonstrated a positive relationship between surfing and wellbeing.

**Conclusions:**

The study provides support that the tool can be a usable and useful process of collecting wellbeing data within surfing. Such a tool may allow us to understand the mechanisms through which surfing and other activities impact wellbeing. However this area of research is still new and more work needs to be done to assess the reliability and validity of the tool.

**Funding Source:**

University of Bristol Grant Capture Fund.

